# Treatment Engagement as a Predictor of Therapy Outcome Following Cognitive Behaviour Therapy for Autistic Children

**DOI:** 10.1007/s10803-023-06083-7

**Published:** 2023-08-29

**Authors:** Carly S. Albaum, Teresa Sellitto, Nisha Vashi, Yvonne Bohr, Jonathan A. Weiss

**Affiliations:** Department of Psychology, York University, 4700 Keele Street, Toronto, ON M3J 1P3 Canada

**Keywords:** Treatment engagement, Cognitive behaviour therapy, Autism, Children

## Abstract

Active engagement in one’s therapy is a key contributor to successful outcomes. Research on child engagement in cognitive behaviour therapy (CBT) has largely focused on youth without autism. This longitudinal study examined multiple indicators of child engagement in relation to outcomes for autistic children who took part in CBT for emotion regulation. **Method:** Data were collected from 60 autistic children who were between 8 and 13 years of age (86.7% male; *M*_*age*_ = 9.58 years, *SD* = 1.44 years; 75% White). Indicators of child engagement included independent observer ratings of in-session involvement, as measured by the *Child Involvement Rating Scale*, and therapist ratings of the therapeutic relationship and homework completion using single-item measures. Indicators of engagement were measured at early (i.e., first third), middle (i.e., mid third), and late (i.e., final third) stages of treatment. Parent-reported emotion regulation was the primary treatment outcome, as measured by the *Emotion Regulation Checklist*. **Results:** After controlling for pre-treatment scores, in-session involvement significantly predicted some aspects of post-treatment emotion regulation, whereas therapeutic relationship and homework completion did not. **Conclusions:** Child in-session involvement throughout therapy may be particularly relevant for treatment change. Addressing issues related to in-session involvement early in treatment may help to promote therapeutic success for autistic children.

**Supplementary Information:**

The online version contains supplementary material available at 10.1007/s10803-023-06083-7.

A growing body of evidence suggests that cognitive behaviour therapy (CBT) may help to reduce mental health challenges for some autistic children (Ameis et al., 2018; Weston et al., 2016). Elevated rates of co-occurring mental health issues in autistic young people are well-documented (Lai et al., 2019; Salazar et al., 2015), and may be related to overarching limits in emotion regulation abilities (Mazefsky & White, 2014); the capacity to monitor, evaluate, and modify emotional responses for the purpose of goal achievement (Thompson, 1994). For example, autistic children demonstrate more intense negative affect during frustrating situations and are more likely to employ maladaptive emotion regulation strategies, such as avoidance and venting, relative to peers without autism (Jahromi et al., 2012). They may also be more emotionally labile due to sensitivity to environmental change, and have difficulty using flexible, adaptive emotion regulation strategies (e.g., altering thoughts to be balanced and realistic) because of core challenges with rigidity (Mazefky & White, 2014). CBT has largely focused on reducing anxiety in autistic youth (Weston et al., 2016), but emotion regulation has also been shown to improve through CBT (Conner et al., 2019; Weiss et al., 2018). Although early findings on the efficacy of CBT for mental health and emotion regulation are promising, it is important to recognize that a notable portion of autistic children who participate in CBT show little to no improvement in their symptoms (Warwick et al., 2017). Research that focuses on the nuances of the therapeutic process may help to identify addressable and modifiable factors that contribute to variation in treatment success.

Active engagement in one’s treatment is a key contributor to successful outcomes (McKay & Bannon, 2004). Child engagement is defined as a therapeutic process involving: *behaviour*, such as participation during and outside of therapy session, and collaboration with the therapist; *cognitions*, such as beliefs about the need for and efficacy of therapy; and *affect*, such as the emotional attitude about therapy (King et al., 2014). Poor treatment engagement in mental health services for youth, in general, is recognized as a “significant public health concern” (Becker et al., 2018). Research on child engagement in therapy is difficult to synthesize due to the inconsistency in how engagement has been operationalized and measured across studies. To help establish consistency in research, Becker et al. (2018) proposed an organizational conceptual framework called *REACH* that outlines key aspects of the broader construct of engagement: **R**elationship (e.g., therapeutic alliance), **E**xpectancy (e.g., beliefs, readiness, or motivation for treatment), **A**ttendance, **C**larity (e.g., understanding about treatment approach and roles), and **H**omework (i.e., in-session involvement and homework completion).

In the literature on youth therapy in general, certain aspects of treatment engagement have been researched more extensively than others. In regard to the *Relationship* component of engagement, there is increasing interest in the role of the therapeutic alliance in child-focused therapies. The most recent meta-analysis of alliance-outcome associations in child psychotherapy identified 28 studies, yielding a small to moderate-sized effect relative to treatment outcome (Karver et al., 2018). The association between alliance and outcome was shown to be moderated by several factors including study design (i.e., RCT vs. non-RCT), presenting problem (e.g., internalizing vs. externalizing), treatment type (e.g., behavioural, non-behavioural), and treatment setting (i.e., inpatient vs. outpatient). Regarding *Homework*, defined in the REACH acronym as participation both within and outside of sessions, a meta-analysis of 13 studies that assessed youth participation in therapy indicated a moderate effect, on average, in relation to treatment outcome (Karver et al., 2006). However, there was large variability in effect sizes across studies, which may be attributed in part to differences in the measures used to assess participation (e.g., parent vs. therapist-report). In a more recent review, Fjermestad et al. (2009) found two studies that measured child involvement using an observational coding scheme (Chu & Kendall, 2004, 2009). Both studies showed small treatment effects for child involvement, with ratings of involvement taken later in therapy having a stronger association with treatment outcome compared to ratings taken at earlier sessions. In terms of the relation between homework completion (i.e., between-session participation) and treatment outcome in CBT for youth, research findings have been mixed. In some studies involving children and adolescents with anxiety, homework completion was not found to be related to treatment change (Arendt et al., 2016; Hughes & Kendall, 2007), whereas other studies found that homework completion predicted clinical improvement for adolescents who participated in therapy to address concerns of depression (Simons et al., 2012), and for children and adolescents with obsessive-compulsive disorder (Park et al., 2014). *Attendance*, one of the main indicators of engagement measured in research on child therapy, has been operationalized in numerous ways, such as the number of sessions attended and/or rates of attrition (Becker et al., 2018). Although attendance is necessary for treatment engagement, attributing engagement to mere presence at sessions fails to capture the *quality* of engagement during sessions, or the relation between engagement and early termination. Other aspects of engagement, including *Expectancy* and *Clarity*, have received almost no attention. The scant research examining child expectancy (i.e., beliefs in the success of, and willingness to participate in therapy) has suggested that although children provide consent to treatment, only a subset of participants indicate interest or motivation for participation (Adelman et al., 1984), and many demonstrate some reluctance or dissatisfaction with their involvement in therapy (Taylor et al., 1985). Interestingly, Karver et al.’s (2006) meta-analysis found only one study on the association between youth *willingness* to participate in treatment and outcome (i.e., Adelman et al., 1984), which indicated a moderate effect size, with no new studies identified since then.

Across the studies described above, there is little to no mention of autistic children, making it unclear whether results generalize to mental health treatment for these youth. As with research in the general child population, the relationship aspect of engagement (i.e., therapeutic alliance) seems to be the most well-studied construct in research involving autistic youth. Findings from several studies suggest that a stronger therapeutic alliance predicts greater symptom improvement following therapy for autistic children and adolescents (Albaum et al., 2020; Brewe et al., 2021; Kerns et al., 2018; Klebanoff et al., 2019). Attendance has been examined in the autism literature as an indicator of treatment feasibility and study quality, focusing mainly on rates of attrition and intervention length (i.e., number of sessions), with little emphasis on the association between attendance and treatment outcome. Compared to youth without autism, autistic youth may engage in therapy in unique ways because of inherent traits (e.g., differences in forming social relationships; concrete thinking style), and common co-occurring challenges in other domains of functioning, such as executive functioning skills and inattention (Demetriou et al., 2018; Salazar et al., 2015). Accordingly, research findings derived from non-autistic samples may not necessarily be generalizable. Establishing separate evidence based on research involving autistic youth is warranted. To our knowledge, there are no studies that have explored other aspects of child engagement, including in-session involvement or homework completion, as well as expectations and clarity about treatment in relation to treatment outcome.

The aim of the present study was to assess multiple indicators of child engagement in relation to CBT treatment outcomes for autistic children using a longitudinal design. Specifically, this study focused on the link between indicators of engagement, including the child-therapist relationship, in-session involvement, and homework completion, and treatment outcome for autistic children who took part in a CBT program that focused on emotion regulation. Indicators of engagement were assessed at early, middle, and late stages of treatment to consider temporal patterns of association that may be overlooked with single time-point data or averaged scores. It was hypothesized that a stronger relationship between child and therapist, greater in-session involvement, and homework completion would predict greater improvements in emotion regulation. Based on findings from the general child literature (e.g., Fjermestad et al., 2009), indicators of engagement measured later in the treatment process were expected to have a stronger association with treatment outcome compared to those measured earlier in treatment.

## Method

### Participants

Participants included 60 autistic children (86.7% male; *M*_*age*_ = 9.58 years, *SD* = 1.44 years, Range: 8–13 years) who were involved in one of two randomized waitlist-controlled trials (RCTs) evaluating the efficacy of CBT for emotion regulation. Children were eligible to take part in the trials if they had a documented diagnosis of autism or related disorder (e.g., Asperger syndrome) made by a licensed healthcare professional, were between 8 and 13 years of age, demonstrated at least average intellectual functioning, and exhibited at least some willingness to take part in therapy (i.e., no remarkable resistance or refusal of treatment). Children were excluded if there were significant concerns regarding disruptive behaviour (e.g., physical aggression, destruction of property) or if the child had an intellectual disability. Based on the *Wechsler Abbreviated Scale of Intelligence*, *2nd Edition* (WASI-II; Wechsler, 2011), IQ for the full sample ranged from 79 to 140 (*M*_*Full−Scale IQ−2*_ = 106.63, *SD* = 14.46). Autism symptom severity, based on the *Social Responsiveness Scale, 2nd Edition* (SRS-2; Constantino, 2012), ranged from Normal to Severe (*M*_*T−score*_ = 73.78, *SD* = 8.98, Range: 51–90). The majority of children were identified by their parents as White (75.0%); 6.7% (*n* = 4) described their ethnicity as “Other” without additional detail, 5.0% (*n =* 3) identified as multi-ethnic, 5.0% identified as Asian (i.e., Chinese, Southeast Asian, West Asian), and 8.3% (*n* = 5) preferred not to disclose their ethnicity. Family household income was over $100,000 CAD for 53.6% of the sample (Note: 17.9% preferred not to disclose household income).

### Measures

#### Child Engagement

For the current study, child engagement was operationalized as: (a) the therapeutic relationship between child and therapist, (b) in-session involvement, and (c) homework completion.

#### Therapeutic Relationship

The therapeutic relationship (TR) between child and therapist was measured using a single item rated by therapists at the end of each session. Therapists were asked “How would you describe the therapeutic relationship with the child?” Ratings were provided on a 7-point Likert-type scale, ranging from 1 (‘Very poor’) to 7 (‘Very good’).

#### In-Session Involvement

In-session involvement (ISI) was measured using the *Child Involvement Rating Scale* (CIRS; Chu and Kendall, 1999), an observational coding scheme assessing child engagement during therapy sessions. The CIRS includes 10 items; six positive and four negative behaviours indicative of treatment involvement: (1) initiating discussion; (2) making suggestions for therapy tasks; (3) demonstrating enthusiasm; (4) self-disclosure; (5) asking the therapist questions; (6) elaborating on therapist’s point or demonstrating understanding; (7) withdrawal or passivity; (8) inhibition or avoidance; (9) distracting away from activities; and (10) oppositionality towards therapist. Each item is rated on a 6-point scale ranging from 0 (‘Not at all present’) to 5 (‘A great deal present’). Negative indicators are reverse-coded, and an overall involvement score is calculated by summing ratings for the ten items. Higher scores indicate more positive in-session involvement. The CIRS was found to have strong internal consistency and interrater reliability in studies involving children without autism who participated in CBT for anxiety (Chu & Kendall, 2004, 2009; Hudson et al., 2014; McLeod et al., 2014) and post-traumatic stress (Ovenstad et al., 2023). This was the first known study to use the CIRS in a sample of autistic children. Internal consistency for the current sample was acceptable for early, mid, and late treatment ratings (Cronbach’s α = 0.62 – 0.74). Information regarding interrater reliability is provided in the Procedure section below.

#### Homework Completion

Homework completion (HC) was reported by the therapist at the end of each session. Therapists were asked to indicate whether participants fully completed (‘2’), partially completed, (‘1’) or did not complete the homework (‘0’) assigned at the previous session. Few participants were rated as having not completed the homework (early sessions, *n* = 7; mid sessions, *n* = 4; late sessions, *n* = 8). Thus, homework completion was subsequently dichotomized as either ‘incomplete’ (i.e., combined scores of 0 or 1) or ‘complete’ (i.e., 2) for the purpose of this study.

#### Treatment Response

The primary treatment outcome for the CBT trials was emotion regulation. Treatment response was assessed using the 24-item *Emotion Regulation Checklist* (ERC; Shields and Cicchetti, 1997). The ERC is a parent-reported measure that assesses effective and ineffective emotion regulation processes displayed by children, comprising two subscales: (1) Lability/Negativity (i.e., emotional reactivity and intensity; dysregulated emotional responses; unstable mood); and (2) Emotion Regulation (i.e., adaptive/effective strategies for managing emotions; stable affect). Responses are provided using a 4-point scale (1 = ‘Never’, 4 = ‘Almost always’), and mean subscale scores are calculated by averaging ratings across subscale items. The ERC was found to have good to excellent internal consistency when used with youth without autism (Shields & Cicchetti, 1997, 1998), and acceptable to good internal consistency in previous research involving autistic youth (Albaum et al., 2020; Weiss et al., 2018).

### Procedure

Secondary analysis of data collected from these trials received approval from the Research Ethics Board at Weiss et al., 2018. For the original RCT, data were collected from 2013 to 2017. Data collection for the second RCT began in 2018, and was discontinued in March 2020 as result of the COVID-19 pandemic. Children who were enrolled in the second RCT but whose participation was impacted by the COVID-19 pandemic were excluded from the current study; only children who completed the intervention and post-treatment assessment prior to the pandemic are included. Study recruitment was done through online advertisements on local autism advocacy websites. Study information was also sent to community care providers (e.g., healthcare professionals, social workers) who were encouraged to share with the families they serve. Families who were interested in taking part in the treatment trial contacted the study coordinator to begin the screening process and determine eligibility. Parents provided written consent and children provided written or verbal assent. Parents also provided written consent to use video-recordings of therapy sessions for research purposes.

Once eligibility was confirmed, families were randomly allocated to either begin treatment the week following baseline assessment or wait 12 weeks after the baseline assessment before starting the program (i.e., waitlist condition). Children in the waitlist condition completed a second assessment following the waiting period, prior to beginning treatment (i.e., pre-treatment score). All children completed a post-treatment assessment within a week following completion of the program. For the purposes of the current study, children in either condition who completed the therapy intervention in its entirety and had post-treatment data available were combined to form one treatment group. Notably, approximately 14% of participants who began the intervention program opted to terminate treatment early, consistent with attrition rates reported in RCTs evaluating CBT (Fernandez et al., 2015). For the original RCT, there were no statistically significant differences between completers and non-completers in regard to demographic and clinical characteristics. Additional details regarding attrition for this trial are available in Weiss et al., 2018.

#### Intervention

Children participated in the *Secret Agent Society: Operation Regulation* (*SAS:OR*; Beaumont, 2013), a CBT program focused on emotion regulation. *SAS:OR* is a 10-session, individual therapy program that is provided on a weekly basis. Sessions are one hour in length, except for the first session, which is 90 min to allow extra time for introductions and rapport building. The child and their primary caregiver are both present for the full session time for all ten sessions. Each session involves homework review, psychoeducation, in-session skill practice, computer games, and planning for home practice. For both trials, *SAS:OR* was provided by post-doctoral fellows and graduate students in clinical and clinical-developmental psychology programs, and were supervised by registered clinical psychologists. Therapists demonstrated acceptable fidelity with the treatment manual (85% ± 11%, Range: 50–100%; Weiss et al., 2018).

#### Measurement Timing

Given the moderation effect that has been documented for measurement timing (i.e., measures taken later in the course of treatment being more strongly associated with outcome compared to measures taken earlier; Chu and Kendall, 2004;, 2009), indicators of child engagement were assessed at the early (i.e., first third), middle (i.e., middle third) and late (i.e., final third) stages of therapy. Measures were not taken from the first or final session as the aims of these sessions (e.g., introducing the child and parent to therapy program, establishing rapport; terminating treatment, planning for the future) tend to differ from the structure and content covered during the intermediary sessions. One early (session 2 or 3), one middle (session 4, 5, or 6) and one late session (session 7, 8, or 9) were randomly selected for each participant. Data for each engagement indicator were based on the same session. For example, therapist ratings of the therapeutic relationship and homework completion, and observational ratings of in-session involvement were all derived from session 2 if this was the session randomly selected as the early-stage session for a participant.

#### Coding Plan

Coders included clinical psychology graduate students (CA, TS, NV) who were familiar with the *SAS:OR* therapy program. Due to the extent of involvement in the larger RCT and the content of each session, coders were unable to be completely masked to participant identity and session number. Training began with in-depth review of the CIRS scoring manual (Chu & Kendall, 1999). Coders reviewed and coded practice sessions together to establish a catalogue of behavioural examples for CIRS item ratings. For reliability training, coders independently coded the same session videos until acceptable interrater agreement was achieved (ICC = 0.87). Once reliability was established, coding methods followed those employed by Chu and Kendall (2009). All three sessions for a given participant (i.e., early, mid, and late) were reviewed and rated by the same coder. Coders watched two 10-minute segments of each video recording, beginning at the 10-minute and then 40-minute marks of the video. For sessions less than 50 min in duration (early sessions, *n* = 5; mid sessions, *n* = 5; late sessions, *n* = 7), the second 10-minute segment began at the 30-minute mark of the video.

Interrater reliability was calculated using a random selection of approximately 30% of coded videos. ICC was computed based on the one-way random effects ICC (1, 1) model, which provides an estimate of reliability for each individual observer’s rating, allowing for the generalization of results to other single observers (Shrout & Fleiss, 1979). To minimize rater drift, ICC was computed at regular intervals during the coding process, and coders met routinely to discuss ICC results and come to consensus on any rating discrepancies. Consensus ratings were used for videos selected for the reliability analysis. Across treatment stages, interrater reliability was consistently good (ICC = 0.82 – 0.88).

### Data Analysis Plan

Demographic and clinical characteristics were examined in relation to indicators of engagement and treatment response using non-parametric bivariate correlations for continuous variables, and independent sample comparisons or Chi-square tests for analyses involving categorical variables. Multiple linear regressions were calculated to assess whether indicators of child engagement predicted treatment response, after controlling for baseline levels of emotion regulation. Statistical significance was evaluated at the alpha < 0.05 level. No adjustments were made to correct for multiple comparisons, as the current study had a small sample size. It was decided that the lack of previous research examining treatment engagement and consideration of significance and relevance of the findings outweighed the risk of increasing a false positive rate (Feise, 2002). Sensitivity analysis using G*Power 3.1 (Faul et al., 2009) indicated that moderate effects could be detected using multiple linear regression analyses including up to five predictors with a sample of 60 children. Data were analyzed using IBM SPSS Statistics version 28.0.0.0 (190). This study’s design and its analysis were not pre-registered.

## Results

### Preliminary Analyses

Descriptive statistics for engagement indicators at each phase of treatment are provided in Table 1, and item-level descriptive statistics for the CIRS are presented in Table 2.

**Table 1:** Descriptive Statistics of Child Engagement Indicators Across Treatment Stages

	Early		Mid		Late	
Indicator	*M*(*SD*)	Range	*M*(*SD*)	Range	*M*(*SD*)	Range
Therapeutic relationship	5.50 (1.03)	2–7	5.88 (1.01)	3–7	5.65 (1.10)	3–7
In-session involvement	24.32 (5.43)	6–40	25.97 (5.89)	13–41	24.02 (5.10)	13–37
Homework completion (% complete)	44.8	–	46.6%	–	59.3%	–

**Table 2:** Descriptive Statistics of CIRS Items

	Early			Mid			Late		
CIRS Item	*M*(*SD*)	*Median*	% rated 4 or 5	*M*(*SD*)	*Median*	% rated 4 or 5	*M*(*SD*)	*Median*	% rated 4 or 5
1. Initiate discussion	0.13 (0.43)	0.00	0.0%	0.35 (0.76)	0.00	0.0%	0.17 (0.62)	0.00	0.0%
2. Make suggestions	0.22 (0.52)	0.00	0.0%	0.47 (0.77)	0.00	0.0%	0.28 (0.61)	0.00	0.0%
3. Demonstrate enthusiasm	2.72 (1.06)	3.00	16.6%	2.88 (1.12)	3.00	23.3%	2.62 (0.98)	2.00	20.0%
4. Self-disclose	1.50 (1.08)	1.00	3.3%	2.07 (1.25)	2.00	11.7%	1.58 (1.37)	1.50	8.3%
5. Ask questions	0.60 (0.98)	0.00	1.7%	0.87 (1.10)	0.50	3.3%	0.53 (0.79)	0.00	0.0%
6. Demonstrate understanding	2.07 (1.04)	2.00	11.7%	1.98 (1.27)	2.00	10.0%	2.23 (1.14)	2.00	13.3%
7. Withdrawal or passivity	0.53 (1.02)	0.00	3.3%	0.35 (0.99)	0.00	3.3%	0.38 (0.80)	0.00	0.0%
8. Inhibition or avoidance	0.55 (1.10)	0.00	3.3%	0.78 (1.15)	0.00	3.3%	0.95 (1.40)	0.00	6.7%
9. Distract	1.37 (1.45)	1.00	10.0%	1.22 (1.43)	1.00	10.0%	1.43 (1.31)	1.00	6.7%
10. Opposition	0.47 (1.16)	0.00	5.0%	0.30 (0.74)	0.00	0.0%	0.63 (1.28)	0.00	8.3%

*Notes*. CIRS = Child Involvement Rating Scale. Possible score range for each item is 0–5. Higher scores for items 1–6 indicate greater involvement, whereas higher scores for items 7–10 indicate less involvement

Raw engagement data were non-normally distributed in terms of either skewness or kurtosis; thus, non-parametric tests were computed to compare scores across treatment phases. Based on related-samples Friedman’s two-way ANOVA by ranks, there were significant differences in therapeutic relationship, *F*_*r*_(2) = 6.93, *p* = .02, and in-session involvement, *F*_*r*_(2) = 7.94, *p* = .03, across therapy stages. Follow-up pairwise comparisons indicated mid-stage in-session involvement was greater than late-stage involvement (*p* = .01); there were no significant differences between early-stage and mid- (*p* = .06) or late-stage scores (*p* = .44). Therapeutic relationship at mid-stage was greater than at early-stage (*p* = .04); there were no significant differences between late-stage and early- (*p =* .60) or mid-stage (*p* = .12) therapeutic relationship. In terms of homework completion, Cochran’s *Q* test determined there were no significant differences in the proportion of youth who completed homework across stages of therapy, *χ*^*2*^(2) = 4.00, *p* = .14. Given the observed differences for some engagement indicators across stages of therapy, subsequent analyses considered engagement scores for each treatment stage separately, instead of aggregating engagement scores across time points. Child clinical factors, including IQ and autism-symptom severity, were not significantly associated with indicators of engagement (all *p*s > 0.05), and child age was not associated with indicators of engagement, except for mid-stage in-session involvement, *r*_*s*_ = 0.27, *p* = .04. Exploratory analyses comparing participants based on randomization allocation (i.e., treatment immediately vs. waitlist control) indicated that there were no significant differences (*p* > .05) between treatment and waitlist participants for early-, mid-, and late-stage in-session involvement and therapeutic relationship. Groups did not differ in terms of early and mid-stage homework completion; however, a greater proportion of treatment participants completed homework at the late stage of therapy compared to waitlist participants, *X*^*2*^ (1, 54) = 4.91, *p* = .03.

### Correlations

Spearman rho correlations between treatment stages for continuous indicators of engagement (i.e., therapeutic relationship and in-session involvement), and partial correlations between indicators of engagement and treatment outcome, controlling for baseline scores, are presented in Table 3.

**Table 3:** Spearman Rho and Partial Correlations Between Continuous Indicators of Child Engagement and Treatment Outcome

	2	3	4	5	6	7^a^	8^b^
1. Early TR	0.25	0.50**	0.22	0.51**	0.24	− 0.26	− 0.13
2. Early ISI	-	0.04	0.64**	0.13	0.52**	− 0.36**	0.20
3. Mid TR		-	0.2^+^	0.62**	0.21	− 0.18	0.19
4. Mid ISI			-	0.11	0.55**	− 0.34*	0.28*
5. Late TR				-	0.36**	− 0.23	0.01
6. Late ISI					-	− 0.28*	0.11
7. ERC Lability/Negativity						-	− 0.31*^a,b^
8. ERC Emotion Regulation							-

*Notes*. ERC = Emotion Regulation Checklist; ISI = In-session involvement; TR = Therapeutic relationship.

** = *p* < .01; * = *p* < .05

^a^ Partial correlation controlling for pre-treatment ERC Lability/Negativity

^b^ Partial correlation controlling for pre-treatment ERC Emotion Regulation

Results of Mann-Whitney *U* tests indicated no significant difference between homework completers and non-completers regarding therapeutic relationship or in-session involvement at each stage of treatment (all *p*s > 0.05). Results of ANCOVA, controlling for baseline scores, indicated no significant differences in post-treatment ERC Lability/Negativity or Emotion Regulation between homework completers and non-completers at early, mid, or late stages of treatment (all *p*s > 0.05; results available in Supplemental Table 1). Since therapeutic relationship and homework completion at each stage of treatment were not significantly associated with the outcome variables, the decision was made to exclude these variables as predictors in subsequent regression models to maximize statistical power.

### Linear Regressions

Details of linear regression results are outlined in Table 4.

**Table 4:** Linear Regression Coefficients for Child Engagement Predicting Treatment Outcome

	ERC Lability/Negativity^a^						ERC Emotion Regulation^a^				
Predictors	*B*	*SE B*	*p*	*sr* ^*2*^	Model Fit		*B*	*SE B*	*p*	*sr* ^*2*^	Model Fit
					*R*^*2*^_*adj*_ = 0.58*						*R*^*2*^_*adj*_ = 0.54
Constant	1.25	0.30	< 0.001				0.91	0.27	0.001		
Baseline	0.70	0.08	< 0.001	0.52			0.60	0.08	< 0.001	0.43	
Early Stage ISI	− 0.01	0.01	0.28	0.01			0.00	0.01	0.64	0.00	
Mid Stage ISI	− 0.01	0.01	0.32	0.01			0.01	0.01	0.22	0.01	
Late Stage ISI	− 0.01	0.01	0.23	0.01			− 0.00	0.01	0.92	0.00	

*Notes.* ERC = Emotion Regulation Checklist; ISI = In-session involvement. *sr*^*2*^ represents semi-partial correlation squared.

^a^ Outcome variable

* *p* < .05

Pre-treatment ERC subscale scores were entered in the first step of the model. Early, mid, and late-stage ISI were simultaneously entered into a second step to examine contribution of overall ISI to post-treatment scores. Regression assumptions were assessed and met for each model. After controlling for baseline levels, overall ISI significantly predicted post-treatment ERC Lability/Negativity scores, *ΔR*^*2*^ = 0.09, *p* = .01. ISI at a single stage of treatment did not uniquely account for portion of variance (all *p*s > 0.05). After controlling for baseline levels, post-treatment ERC Emotion Regulation was not significantly predicted by overall ISI, *ΔR*^*2*^ = 0.04, *p* = .24, and unique variance was not accounted for by ISI at any single stage of treatment.

## Discussion

Focusing on nuances of the therapeutic process can edify what is presently known about the usefulness of CBT for autistic children. Treatment engagement is considered an essential aspect of the therapy process (McKay & Bannon, 2004), but there is a lack of empirical research examining engagement in CBT for youth, and even less attention paid in research on CBT for autistic children. This study evaluated indicators of treatment engagement in relation to treatment outcome for autistic children who took part in a CBT program focused on emotion regulation. Indicators of treatment engagement were selected based on Becker et al.’s (2018) conceptual framework, *REACH*, concentrating on (a) the therapeutic relationship between child and therapist, (b) in-session involvement, and (c) homework completion, as dimensions of engagement (for full details regarding the REACH framework, see Background). Engagement was measured at early (i.e., first third of treatment), mid (i.e., middle third), and late (i.e., final third) stages of therapy to consider the potential influence of measurement timing that has been documented in research involving children without autism (e.g., Chu and Kendall, 2004;, 2009). It was expected that greater engagement would predict better post-treatment emotion regulation, after controlling for pre-treatment levels, and that indicators measured later in therapy would have stronger associations with outcome compared to indicators measured earlier in therapy.

In partial support of hypotheses, overall in-session involvement was predictive of the change found in emotional lability and negativity (a major target of the intervention) by the end of treatment, with a moderate-sized effect in relation to post-treatment improvement. In contrast, in-session involvement did not predict improvements in adaptive regulation improvements post-treatment, as measured by the Emotion Regulation subscale of the ERC. The difference in the pattern of results between the ERC Lability/Negativity and Emotion Regulation subscales may be attributed in part to differences in change that occur in maladaptive compared to adaptive emotion regulation processes over the course of therapy, within the context of the intervention program being evaluated. Although not directly comparable, Chu and Kendall (2004) found similar effect sizes when examining whether in-session involvement in CBT was associated with reductions in anxiety for youth without autism. The authors reported that in-session involvement during the second quarter of treatment accounted for 8% of variance in anxiety symptom change, relative to the approximate 9% observed for overall in-session involvement in the present study. The involvement-outcome association is also somewhat comparable to meta-analytic findings from the general child literature that indicate a small-sized correlation between youth participation and treatment outcome (Karver et al., 2006), suggesting that in-session involvement may be as relevant, if not more, for treatment change for autistic children as it is for those without autism.

Current findings suggest autistic youth demonstrate both positive and negative behaviours indicative of involvement during therapy sessions. Both the present study and Chu and Kendall (2004) examined the positive characteristics of involvement that are pertinent to the therapeutic process for child-focused therapy (Braswell et al., 1985), including initiating discussions related to session activities and goals, demonstrating enthusiasm in therapy-related tasks, self-disclosing relevant information that does not attempt to distract from the focus of the session, and demonstrating understanding of the therapeutic skills. Research involving adult clients suggests that *negative* aspects of involvement, such as hostility or negative reactions towards the therapist, may also be related to treatment outcome (Gomes-Schwartz, 1978; O’Malley et al., 1983). Initial findings using the CIRS indicated that negative externalizing behaviours, such as oppositionality and diversion from session tasks, seldomly occurred in therapy for anxiety in youth without autism (Chu & Kendall, 2004). Due to the infrequent occurrence of these behaviours and concerns regarding the validity of negative items, subsequent studies have excluded these items from analyses when using the CIRS with samples of youth predominantly impacted by internalizing problems (e.g., Chu and Kendall, 2009; Hudson et al., 2014). However, given the common occurrence of oppositional and attentional challenges (Salazar et al., 2015), and emotion dysregulation (Mazefsky & White, 2014) demonstrated by autistic youth, items examining these negative aspects of involvement were included for the current study. Indeed, autistic youth who participated in the current intervention varied in the extent to which they demonstrated oppositionality towards therapist, and engaged in off-task behaviour that diverted away from the focus of the session. Previous research suggests that in practice, therapist flexibility is key for adapting therapy for autistic people (Kerns et al., 2016; Spain & Happé, 2020), and may be an essential skill for promoting positive involvement and minimizing disruptive behaviour that can impede therapeutic progress. Therapists that work flexibly to adapt treatment to accommodate child needs are more likely to foster positive client involvement, in turn contributing to more successful outcomes (Chu & Kendall, 2009). Future research should aim to empirically evaluate the association between therapist flexibility, child in-session involvement, and treatment outcomes for autistic youth to better understand the transactional nature of the therapeutic process for this population.

There were minimal differences between therapy stages (i.e., early, middle, late) on the strength of associations between in-session involvement and outcome. The only other known study using the CIRS in individual CBT reported that later-stage in-session involvement was a predictor of treatment outcome (i.e., anxiety reduction) for children without autism, while early stage was not (Chu & Kendall, 2004). In their study, the measurement of involvement in the ‘late’ treatment stage was assessed during the second quarter of therapy (i.e., still within first half); involvement was not measured during the second half of treatment, meaning that they only assessed early and middle stages of treatment, and did not examine the actual late stage. Researchers who have used the CIRS to assess involvement relative to other therapeutic process factors for children without autism have found that child involvement tends to peak around the middle of treatment (Hudson et al., 2014). Taken together, results from these studies suggest involvement in the mid-stage of therapy may be the strongest predictor of treatment outcome for youth without autism, compared to measurements taken earlier or later in therapy. However, findings from the current study indicate in-session involvement *throughout* therapy may contribute to treatment change for autistic youth. It may be particularly important for therapists to ensure that issues with involvement are addressed early enough in treatment, such that autistic children are able to participate to the best of their ability for the remainder of therapy. In cases where youth are less involved in the early stages, therapists should continue to encourage participation, as there may be potential for involvement even late in therapy to influence treatment outcomes.

Contrary to hypotheses, the therapeutic relationship was not found to be significantly related to emotion regulation. These findings are inconsistent with previous research that has examined therapeutic alliance between therapists and young autistic clients (Albaum et al., 2020; Kerns et al., 2018; Klebanoff et al., 2019). Therapeutic alliance, as reported by therapists, has been shown to predict reduction in anxiety symptoms (Klebanoff et al., 2019) and global symptom severity (Kerns et al., 2018). In addition, specific aspects of the therapeutic alliance (i.e., task collaboration), as rated by independent observers, have been found to predict improvements in emotional lability and negativity following CBT (Albaum et al., 2020). Notably, studies that found a significant alliance-outcome association employed more rigorous measures of therapeutic alliance that ask respondents about specific minutiae of the relationship, compared to the single global item used for the current study. Using multi-item measures may encourage therapists to reflect more deeply on the relationship, and can help ensure therapists take the various theoretical components of therapeutic alliance into account (i.e., task collaboration; therapeutic bond; goal agreement), which may be overlooked when describing the relationship globally on a single item. This may be particularly important when comparing the predictive nature of alliance alongside other measures of engagement, such as involvement, which was measured in the present study in a much more robust manner. In addition to therapist-reports, future research may also incorporate multi-informant measures of therapeutic alliance to capture the subjective perceptions of the relationship, as experienced by autistic youth and their parents.

Homework completion was also not found to be related to treatment outcomes. Almost all the children in the current sample were rated by therapists as having completed between-session homework at least partially, and differences in treatment change between those who partially versus fully complete homework may be unnoticeable. To our knowledge, there are no other studies that have examined the relation between homework completion and therapeutic success in CBT for autistic youth. Results are consistent with some research involving children without autism, which also found no relation between homework completion and reductions in anxiety (Arendt et al., 2016; Hughes & Kendall, 2007). For example, following 16 sessions of CBT, average homework compliance, as rated by therapists on a single item at the end of each session, (i.e., “Rate the child’s degree of compliance with the homework task.”) was not found to predict clinician-rated principal anxiety disorder severity (Hughes & Kendall, 2007). In a more recent study, parent- and youth-reported homework adherence, operationalized as average time spent each day completing session homework, also did not predict improvements in clinician-rated or child self-reported anxiety symptoms at the end of therapy (Arendt et al., 2016). In contrast, Park et al. (2012) found that increased therapist-rated homework compliance predicted reduced clinician-rated obsessive-compulsive symptom severity. Therapists in this study were asked to consider both quantity *and* quality of homework compliance for exposure-based assignments. For example, therapist ratings considered the difficulty of exposure completed, whether exposure to feared stimuli was accidental or deliberate, and the effort put forth by the child in completing the assigned homework. Therefore, it may be important to measure factors beyond mere completion, such as degree of ease or difficulty of homework, concerted effort in completing homework, and client feelings about homework. Although findings of the current study suggest measurement timing may not be particularly relevant when considering homework completion (when most children are rated to either fully or partially complete their homework across stages), tracking homework completion in this more nuanced manner may provide clinicians with insight into other aspects of engagement and treatment progress.

### Limitations

Results should be interpreted within the context of the study limitations. Several methodological limitations are important to consider. Firstly, the sample only included children who completed treatment in its entirety. Attrition is a key issue in intervention research generally because of the contribution to reduced sample size (Nock & Ferriter, 2005), but may be even more relevant for research focused on treatment engagement, as there may be pertinent differences between those who complete treatment and those who terminate prematurely in regard to engagement-related factors. Relatedly, small sample size is a ubiquitous issue in psychological research because of the connection to statistical power and the capacity to detect true small-sized effects (Marszalek et al., 2011). Within the present study, *p*-values corresponding to several small-sized correlations (e.g., association between therapeutic relationship and post-treatment lability/negativity) did not exceed the threshold for statistical significance (i.e., *p* < .05), which can likely be attributed in part to the small sample size. At the same time, no alpha-level adjustments were made to correct for multiple comparisons, which should be taken into consideration when interpreting statistical results. Coders were not completely masked to session number or participant identity, which introduces risk of bias. Measurement tools employed in this study were either developed using samples of children without autism (i.e., CIRS), or were prospectively chosen to assess treatment feasibility in the clinical trials from which the current study was derived (i.e., therapist ratings of homework completion and therapeutic relationship). Measure development and construct validation that incorporate an autistic lens should be research priorities to ensure measures are appropriate for use with autistic youth (e.g., language is comprehensible, direct, and specific), and to enhance the validity, quality and replicability of research on treatment engagement in CBT for this population (Raymaker & Nicolaidis, 2013). In addition, the present study focused on some, but not all aspects engagement. Future research should consider other indicators, such as treatment expectations, attendance, and clarity, as described by Becker et al. (2018), as well as aspects of parent involvement (Albaum et al., 2023) to generate a more holistic understanding of treatment engagement. Finally, there was a lack of diversity in the sample in terms of ethnicity and socioeconomic status, and a restricted range in terms of intellectual functioning. Researchers should deliberately aim to recruit culturally diverse samples and include autistic children with co-occurring intellectual disabilities. Future research may also consider examining demographic (Wintersteen et al., 2005) and perhaps neurotype match between client and therapist (Crompton et al., 2020) in relation to indicators of engagement.

## Conclusions

Treatment engagement is a fundamental consideration in determining whether autistic children will benefit from CBT. Child in-session involvement throughout therapy may be particularly relevant for treatment change. The therapeutic relationship and homework completion should continue to be considered and evaluated in relation to treatment outcomes to better understand the relevance of these factors in CBT for autistic youth. Future research should aim to develop psychometrically sound measures that assess treatment engagement, while taking into consideration the differences in behavioural, emotional, and social functioning displayed by autistic children compared to those without autism. Researchers should also aim to conceptualize and assess treatment engagement in a consistent way to allow for replication and comparability of findings across studies. To continuously monitor treatment engagement, clinicians may find it useful to implement brief post-session surveys assessing youth or parent perceptions of the therapeutic relationship, the relevance of session goals or topics, and homework. Addressing issues related to in-session involvement early in treatment would likely be beneficial for promoting positive engagement from autistic clients for the remainder of therapy, in turn increasing the likelihood of therapeutic success.

## Electronic supplementary material

Below is the link to the electronic supplementary material.

Supplementary Material 1

## Data Availability

This study’s data have not been made publicly available as participants did not consent to affording anyone beyond the research team with access to the data. The Research Ethics Board at Weiss et al., 2018. has indicated data cannot be made openly accessible without explicit consent from participants. Other research materials and analysis code are available upon request.
