# Supplementary material for: Treatment Engagement as a Predictor of Therapy Outcome Following Cognitive Behaviour Therapy for Autistic Children
Source: J Autism Dev Disord. 2023 Aug 29;54(10):3575–86. doi: 10.1007/s10803-023-06083-7 (PMC11461639; doi:10.1007/s10803-023-06083-7)
Supplement: Supplementary file 1 — Supplementary Material 1 [file 10803_2023_6083_MOESM1_ESM.docx]

| **Supplemental Table 1**  *Comparison of treatment outcomes between homework completers versus non-completers controlling for baseline* | | | | |
| --- | --- | --- | --- | --- |
|  | Non-Completers | Completers |  |  |
|  | *M* (*SE*)^a^ | *M* (*SE*)^a^ | *F*(*df*) | *p* |
| Early stage HC (%) | 55.2% | 44.8% |  |  |
| ERC Lability/Negativity | 2.24 (0.06) | 2.20 (0.06) | 0.14 (1, 53) | .71 |
| ERC Emotion Regulation | 3.01 (0.05) | 3.02 (0.06) | 0.01 (1, 53) | .93 |
| Mid stage HC (%) | 53.4% | 46.6% |  |  |
| ERC Lability/Negativity | 2.26 (0.06) | 2.17 (0.06) | 1.30 (1, 53) | .26 |
| ERC Emotion Regulation | 3.02 (0.05) | 3.01 (0.06) | 0.001 (1, 53) | .98 |
| Late stage HC (%) | 40.7% | 59.3% |  |  |
| ERC Lability/Negativity | 2.32 (0.07) | 2.16 (0.05) | 3.58 (1, 49) | .06 |
| ERC Emotion Regulation | 2.97 (0.06) | 3.07 (0.05) | 1.63 (1, 49) | .21 |
| *Notes*. ERC = Emotion Regulation Checklist; HC = Homework completion.  ^a^ Estimated marginal means controlling for pre-treatment score | | | | |
